# Validating a Reading Assessment Within the Cognitive Diagnostic Assessment Framework: Q-Matrix Construction and Model Comparisons for Different Primary Grades

**DOI:** 10.3389/fpsyg.2021.786612

**Published:** 2021-12-16

**Authors:** Yan Li, Miaomiao Zhen, Jia Liu

**Affiliations:** ^1^Beijing Key Laboratory of Applied Experimental Psychology, Faculty of Psychology, Beijing Normal University, Beijing, China; ^2^Tsinghua Laboratory of Brain and Intelligence, Department of Psychology, Tsinghua University, Beijing, China

**Keywords:** cognitive diagnostic assessment, cognitive diagnostic models, G-DINA, Q-matrix validation, reading comprehension assessment, primary students

## Abstract

Cognitive diagnostic assessment (CDA) has been developed rapidly to provide fine-grained diagnostic feedback on students’ subskills and to provide insights on remedial instructions in specific domains. To date, most cognitive diagnostic studies on reading tests have focused on retrofitting a single booklet from a large-scale assessment (e.g., PISA and PIRLS). Critical issues in CDA involve the scarcity of research to develop diagnostic tests and the lack of reliability and validity evidence. This study explored the development and validation of the Diagnostic Chinese Reading Comprehension Assessment (DCRCA) for primary students under the CDA framework. Reading attributes were synthesized based on a literature review, the national curriculum criteria, the results of expert panel judgments, and student think-aloud protocols. Then, the tentative attributes were used to construct three booklets of reading comprehension items for 2–6 graders at three key stages. The assessment was administered to a large population of students (*N* = 21,466) in grades 2–6 from 20 schools in a district of Changchun City, China. Q-matrices were compared and refined using the model-data fit and an empirical validation procedure, and five representative cognitive diagnostic models (CDMs) were compared for optimal performance. The fit indices suggested that a six-attribute structure and the G-DINA model were best fitted for the reading comprehension assessment. In addition, diagnostic reliability, construct, internal and external validity results were provided, supporting CDM classifications as reliable, accurate, and useful. Such diagnostic information could be utilized by students, teachers, and administrators of reading programs and instructions.

## Introduction

Many existing learning and assessment systems generate scores, levels, and ranks to evaluate students’ learning outcomes. This single outcome evaluation form has caused many problems, such as hurting students’ self-esteem, heightening excessive competition, and increasing the learning burden, which are not conducive to the overall development of students (Lei, 2020). Therefore, new approaches are needed to improve outcome evaluation in the stage of basic education by keeping the evaluation content consistent with the curriculum criteria, providing diagnostic information on students’ strengths and weaknesses in learning, and offering evidence for schools to implement intervention measures.

Cognitive diagnostic models (CDMs) are confirmatory latent class models that combine cognitive theory and psychometric models to reveal the innate structure of a given ability by estimating an individual’s knowledge and skill mastery state (Leighton and Gierl, 2007). CDMs can group examinees into similar latent classes and thus can compensate for the deficiency of single outcome results generated*via* classical test theory and traditional item response theory (Ravand and Robitzsch, 2018). Due to the need for formative evaluation and instructions, CDMs have become popular in educational settings. However, Ravand and Baghaei (2020) noted that over 95% of CDM studies are methodological or simulation-oriented, approximately 4% are retrofitting, and less than 1% focus on real diagnostic test development in recent decades. Therefore, real CDM application studies have rarely found their ways into educational systems, probably because of the lack of reliability and validity evidence and thus limited confidence in the information provided by CDMs (Sessoms and Henson, 2018). There is still a wide gap between CDMs and educational practices, and true CDM studies to develop diagnostic tests from scratch are urgently needed (Alderson, 2010; Sessoms and Henson, 2018; Ravand and Baghaei, 2020).

### CDA Framework

One of the ultimate purposes of CDMs is to make inferences about what attributes an examinee has mastered using a diagnostic assessment. That is, CDA offers valuable information on the diagnostic quality of test items as well as the skill mastery patterns of test-takers, classifying those who have not mastered the item’s required skills, named non-masters, as distinct from those who have, named masters. The CDA frameworks have been proposed and optimized since Rupp and Templin (2008) published the first didactic introduction (de la Torre and Chiu, 2016; Ravand and Baghaei, 2020). In general, the construction of CDA depends on two major elements: the implicit theory section and the CDM section.

The first step in CDA is to specify the implicit attributes that a test-taker must possess to solve an item. The generic term “attribute” is defined as posited knowledge and thinking skill (de la Torre and Douglas, 2004) or a description of the processes, subskills, and strategies that are vital for the successful execution of a particular test (Leighton et al., 2004). Once the target attributes are defined *via* domain experts or think-aloud protocols, individual test items can be coded at the point of item development as a Q-matrix, an incidence matrix that transforms cognitive attributes into observable item response patterns (Tatsuoka, 1990; Li, 2011). It is essential to point out that diagnostic feedback is valid only when the attribute specification is complete, the items effectively measure the targeted attributes, and the attributes are correctly specified in the Q-matrix (Ravand and Baghaei, 2020). The quality of inferences about students is unlikely to be ensured in retrofitting studies, as they commonly include items that fail to adequately tap specific cognitive characteristics (Gierl and Cui, 2008; Chen and de la Torre, 2014).

Then, CDMs are utilized to group examinees with similar skill mastery profiles, to evaluate the diagnostic capacity of items and tests and thus to reveal the degree to which they can measure the postulated attributes (Ravand and Robitzsch, 2018). CDMs make various assumptions to reveal the innate structure of a given ability by estimating the interactions among attributes (Leighton and Gierl, 2007). That is, representative CDMs can mainly be classified into three types: compensatory, non-compensatory, and general models. In compensatory CDMs, mastering one or more targeted attributes can compensate for other attributes that are not mastered. The deterministic input noisy-or-gate model (DINO; Templin and Henson, 2006) and the additive CDM (A-CDM; de la Torre, 2011) are the most representative compensatory CDMs. In contrast, if an attribute has not been mastered, the probability of a correct response in the non-compensatory CDM would be low, as other mastered attributes cannot fully compensate for it. Representative non-compensatory CDMs include the deterministic input noisy-and-gate model (DINA; Haertel, 1989) and the reduced reparameterized unified model (R-RUM; Hartz, 2002). General CDMs allow the estimation of both compensatory and non-compensatory interactions among attributes within the same test, which has influentially led to the unification of various CDMs. The most famous general model is the general DINA model (G-DINA; de la Torre, 2011), which can be transformed into the abovementioned CDMs simply by setting specific constraints to zero or changing link functions.

Like other statistical models, a CDM has no value if it fits the data poorly (de La Torre and Lee, 2010). Specifically, the fitness of CDMs can be ascertained in two ways. Relative fit indices evaluate whether the fit of one model differs significantly from that of another, and the model with smaller relative fit values is judged to better fit the data (Lei and Li, 2016). According to previous research, three well-known relative fit indices are also applicable to CDM studies, including −2 log-likelihood (−2LL), Akaike’s information criterion (AIC), and Bayesian information criterion (BIC; Lei and Li, 2016). In addition, absolute fit indices examine the adequacy of a single model (Liu et al., 2017). For instance, a model can be considered a good fit only if the value of the standardized root mean square residual (SRMSR) is less than 0.05 (Maydeu-Olivares, 2013; George and Robitzsch, 2015). In addition, the max χ^2^, which is the mean of the χ^2^ test statistics of independence for all item pairs, was found to be sensitive in specifying model misfit (Chen and Thissen, 1997; Lei and Li, 2016). A significant value of *p* of max χ^2^ suggests that the model fits poorly (George and Robitzsch, 2015).

### CDM Applications in Reading Tests

As one of the most frequently assessed skills, reading is considered a prerequisite for success in school and life (Kim and Wagner, 2015). As complex and multiple-task abilities, the innate characteristics of reading comprehension have been widely discussed (Barnes, 2015). For example, the construction-integration model regards reading as a meaning-construction process that involves interaction between both reader and text and is influenced strongly by background knowledge (Kintsch, 1991; Snow, 2002). This model characterized reading as an iterative and context-dependent process by which readers integrate information from a text (Compton and Pearson, 2016). In contrast, theorists of component models have pointed out that some important language knowledge, cognitive processes, and reading strategies make relatively independent contributions to reading comprehension (Cain et al., 2004; Cain, 2009). These models indicate that subcomponents of reading, including but not limited to vocabulary, syntax, morphology, semantics, inference, reasoning, discourse comprehension, working memory, and comprehension monitoring, are strong and persistent predictors for readers from children to adults (Aaron et al., 2008; Kim, 2017). Although many studies found that Chinese reading and English reading shared significantly in common (Mo, 1992; Chen et al., 1993), a consensus has not been reached on the number of subcomponents involved at different developmental stages. For example, Mo (1992) proposed that the structure of Chinese language reading displayed a “replacing developmental pattern.” Factor analysis results of a reading test battery suggested that 75% of the variance in grade-6 students’ reading ability was explained by six factors, including word decoding, integration and coherence, inference, memory and storage, fast reading, and transfer ability. As grades increased to the secondary and high school levels, the influences of the abovementioned factors remained important but were partly replaced by newly emerged, higher-level factors such as generalization ability, evaluation ability, and semantic inference ability.

Early research on reading cognitive diagnosis tried to explore the separability of reading ability and identify whether there are relatively independent cognitive components, processes, or skills in reading ability. For example, Jang (2009) found that evidence in Markov chain Monte Carlo aggregation supported the separability of reading into 9 attributes, and most LanguEdge test items have good diagnostic and discrimination power to measure the attributes well. Then, CDMs have been applied to retrofit the data of large-scale reading assessments such as the Progress in International Reading Literacy Study (PIRLS), the Programme for International Student Assessment (PISA), the Test of English as a Foreign Language (TOEFL), the Michigan English Language Assessment Battery (MELAB), and the Iranian National University Entrance Examination (e.g., Jang, 2005; Sawaki et al., 2009; Li, 2011; Chen and de la Torre, 2014; Chen and Chen, 2016; Ravand, 2016; Ravand and Robitzsch, 2018; Javidanmehr and Sarab, 2019; George and Robitzsch, 2021; Toprak-Yildiz, 2021). Many studies have used one preset CDM for reading tests, including DINA (George and Robitzsch, 2021), Fusion (Jang, 2009; Li, 2011), LCDM (Toprak and Cakir, 2021), or G-DINA (Ravand, 2016) models. Only a few compared multiple CDMs and found that general models, such as G-DINA or LCDM, had better fits for reading assessment data (Chen and Chen, 2016; Li et al., 2016; Ravand and Robitzsch, 2018; Javidanmehr and Sarab, 2019). In some cases, compensatory models such as A-CDM or LLM have shown a relatively close fit to those of general models (Li et al., 2016; Chen and de la Torre, 2014). Therefore, researchers called for further comparison of general and reduced CDMs for optimal performance and for an understanding of the interaction mechanism among reading attributes.

In the context of real CDA applications in reading assessment, research is relatively scarce. One notable effort was conducted by Xie (2014), in which a reading comprehension assessment of modern Chinese prose for junior high school students was developed and validated. Fusion model results revealed an unstructured attribute hierarchy of Chinese reading, which was composed of word decoding, formal schema, information extraction, information deduced, content analysis, content generalization, and text evaluation. In addition, Toprak and Cakir (2021) examined the second language reading comprehension ability of Turkish adults with a cognitive diagnostic reading test using the CDA framework.

We collected a total of 15 relevant empirical reading studies in diverse age groups with various language backgrounds and summarized a list of candidate attributes (see Supplementary Table 1 for details) and CDMs for the next phases of test development and analysis. This detailed review yielded 6 commonly specified cognitive attributes, including vocabulary, syntax, retrieving information, making inferences, integration, and evaluation. Text-related attributes, such as narrative text, expository text, and discontinuous text, were also specified in studies of PIRLS and PISA. However, the abovementioned large-scale reading assessments were generally designed and developed under a unidimensional item response theory approach. CDM implementations to extract diagnostic feedback may raise severe issues with model fit, item characteristics, and diagnostic inferences for retrofitting data (Rupp and Templin, 2008; Gierl et al., 2010; Sessoms and Henson, 2018).

Primary students are in the key stages of reading development, during which they need to transition from “learning to read” to “reading to learn,” and begin to encounter difficulties in new comprehension requirements (Carlson et al., 2014). The need for suitable instructions and reading materials as scaffolding is felt mostly at the primary level; therefore, assessing the extent to which the reading ability and subskills of students grow is valuable during their primary school years. However, students’ reading ability grows so much over the course of their schooling that a single-booklet testing design for all grades is beset with problems (Brennan, 2006). Multilevel booklet designs are typically adopted, of which the contents and difficulty can be purposefully differed to balance test precision and efficiency. However, to the best of our knowledge, all CDM implementations were conducted on a single reading booklet for second language learners or grade 4 students and above. Several authors (e.g., Ravand, 2016; Sessoms and Henson, 2018) have briefly noted that CDM applications might be specific to different characteristics of items or students. The construct equivalence of reading attributes and the generalizability of CDMs to other key developmental stages of reading remain unproven.

To address these issues, this study had three goals: (a) to illustrate how the cognitive diagnostic assessment (CDA) framework can be applied to develop the Diagnostic Chinese Reading Comprehension Assessment (DCRCA) for primary students at various key stages, (b) to evaluate the attribute equivalence and model fit adequacy of the CDMs for different developmental stages, and (c) to validate the diagnostic inferences of the DCRCA about primary students’ reading subskills. To answer these questions, the study was mostly concerned with the construction of cognitive models of Chinese reading, the model-data fit evaluation of CDMs for three reading booklets, the validation of diagnostic psychometric properties, and the skill mastery profiles of primary students. This process can shed light on the limited CDA applications in reading test development and provide new methodologies for exploring reading skill structure. To the best of our knowledge, this is the first reading assessment whose CDM model fitness, diagnostic reliability and validity were examined at various developmental stages.

## Materials and Methods

**Figure 1:**
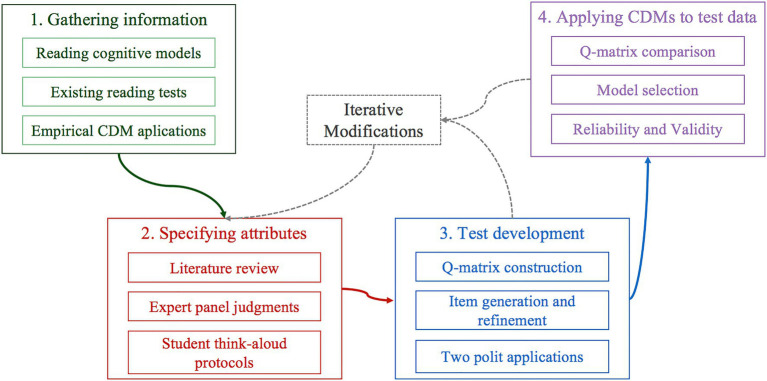
An overview of the research processes.

The development and validation of the reading assessment followed the guidelines of the CDA framework (Ravand and Baghaei, 2020). The research processes are outlined in Figure 1.

### Attributes Specification

Reading attributes were specified through multiple steps, involving domain experts and test-takers who participated in the determination of the core reading features for further curricular use.

- **Literature review**: Candidate attributes were summarized by reviewing 15 empirically validated studies (see Supplementary Table 1), particularly based on those of Chinese reading and native language reading of primary students (Xie, 2014; Yun, 2017; George and Robitzsch, 2021; Toprak-Yildiz, 2021). This detailed review yielded 6 commonly specified cognitive attributes, including retrieving information, making inferences, integration and summation, evaluation, vocabulary, and syntax, as well as three text-related attributes, including narrative text, expository text and discontinuous text.
- **Expert panel’s judgments**: As reading attributes are highly dependent on the characteristics of Chinese reading and the framework of reading education, researchers invited five experts in reading assessment or education to obtain their judgments of large-scale reading assessments and the Chinese Language Curriculum Criterion for Compulsory Education (abbreviated as the curriculum criterion). The “syntax” attribute was first excluded because the curriculum criterion does not advocate any grammar teaching or evaluation at the primary school level but emphasizes helping students comprehend naturally occurring materials in a real language environment (Ministry of Education, 2011). Vocabulary is considered as important as reading comprehension at the primary level, and therefore, this skill was excluded and evaluated by the Chinese Character Recognition Assessment in the test battery. Infrequent attributes were also discussed case by case. For example, formal schema (Xie, 2014) was excluded because it might blend text evaluation with text-type attributes. The importance of literary text (i.e., narrative text and poetry) at the primary level has been emphasized by the curriculum criterion as well as large-scale assessments, including the PIRLS and PISA. However, inconsistencies in other text types have been observed. The curriculum criterion merges expository text (extracted from PIRLS) and discontinuous text (extracted from PISA) into practical text, as they have similarities in their reading objectives and strategies (Compulsory Education Curriculum and Textbook Committee of the Ministry of Education, 2012). After discussion, all experts agreed that this inconsistency was worth further evaluation *via* empirical results.
- **Student think-aloud protocols**: To clarify the cognitive procedures that test-takers went through, 15 students from grades 2 to 6 were selected for think-aloud protocols. These students verbalized their thoughts when solving sample items. According to their answers and oral explanations, researchers identified clues to cognitive processes with an eye on the attributes inferred from the previous procedures. Overall, researchers specified and defined an initial set of eight attributes that might be crucial for primary school students (Table 1).

**Table 1:** Definitions of the initial reading attributes.

No.	Attribute	Definition
α_1_	Retrieving information	Retrieving information requires the abilities to understand a text literally and match the micro/macrolevel propositions to relevant parts of the text (Kintsch, 1991; O’Reilly and Sheehan, 2009; Xie, 2014).
α_2_	Making inferences	Making inferences require combining reader background knowledge with contextual clues to determine implicit meaning and form a beyond surface-level understanding of the text (van Dijk and Kintsch, 1983; Toprak and Cakir, 2021).
α_3_	Integration and summation	Integration and summation require an understanding of relationships across sentences and paragraphs as well as an understanding of the comparative importance of information (main and supporting; Grabe, 2009; O’Reilly and Sheehan, 2009).
α_4_	Reflective evaluation	Reflective evaluation requires an understanding of the author’s purpose, mood, tone, and stance toward the subject as well as evaluating the quality or appropriateness of a text (Chen and de la Torre, 2014; Xie, 2014; Toprak and Cakir, 2021).
α_5_	Literary text	Literary text includes stories, folktales, legends, fables, simple fiction, nursery rhymes, narrative poem, limerick, and shallow ancient poetry (Common Core State Standards Initiative, 2010; Ministry of Education, 2011).
α_6_	Practical text	Practical text contains shallow expository text and discontinuous text at the primary school level (Common Core State Standards Initiative, 2010; Ministry of Education, 2011).
α_6a_	Expository text	Expository text includes illustrative text and simple argumentative text (Yun, 2017).
α_6b_	Discontinuous text	Discontinuous text displays digital sources on a range of topics and information in charts, graphs, or maps (Chen and Chen, 2016).

### Test Development

According to the curriculum criterion, reading education can be divided into three key stages at the primary level. Key stage one is for grades 1 to 2, key stage two is for grades 3 to 4, and key stage three is for grades 5 to 6. Therefore, three booklets of reading diagnosis items were compiled for students at each key stage. An initial common Q-matrix for the three booklets was intentionally designed, as each item reflects one of the four cognitive processes of reading comprehension (α_1_–α_4_) and one text-related attribute (α_5_, α_6a_, and α_6b_). The genre and complexity of texts were controlled, as they were important factors in assessing reading comprehension (Collins et al., 2020). Fragments of literary texts (including fairy tales, stories, fables, narratives, novels, and children’s poems) and practical texts (including explanatory texts, simple argumentative articles, and discontinuous texts) were carefully selected and modified as item stems. A Chinese readability formula (Liu et al., 2021) was adopted to calculate the length, token types, lexical difficulty, function word ratio, and overall difficulty of each text. The average text length of the three booklets ranges from 150.60 to 278.57 characters, and the average text difficulty levels for the three booklets are 3.38, 3.69, and 4.40 (for details, please see Supplementary Table 2). Therefore, the three booklets are composed of conceptually appropriate short texts with increased complexity.

The item generation procedures were as follows: mapping cognitive and text-type attributes to compile 73 draft multiple-choice items, an expert review to cross-validate the Q-matrix, and item refinement following the expert review. Then, after the first pilot using two booklets for grade 1–2 and 3–6 students (*n* = 378), 17 problematic items were removed according to the item discrimination index (item-total correlation <0.19), and several items were modified. Grade 1 students were excluded from further study because they could not adapt to the computer assessment procedures. The second pilot included 56 items in three booklets, and each booklet consisted of 18–20 items. Pilot data were obtained from 5,949 grade 2–6 students. Both classical test theory and a 2PL item response model analysis were conducted. Five items with unsatisfactory discrimination (item-total correlation <0.30 or IRT discrimination <0.50) and three items with moderate to large differential item functioning issues on gender (effect size >0.88) were removed. A total of 48 items were retained, and four items were modified or rearranged for facility (passing rates by grade < 0.20 or > 0.90). The four cognitive attributes were intentionally balanced in testing frequency (4 to 5 times each attribute), and the proportion of literacy and practical texts were similar in the three booklets. Therefore, as shown in the last line of Table 2, the total testing frequencies of the attributes were similar in the three final booklets, with slight differences in item order and proportions of text type.

**Table 2:** Initial Q-Matrices.

Item	Booklet KS1								Booklet KS2								Booklet KS3							
	α_1_	α_2_	α_3_	α_4_	α_5_	α_6_	α_6a_	α_6b_	α_1_	α_2_	α_3_	α_4_	α_5_	α_6_	α_6a_	α_6b_	α_1_	α_2_	α_3_	α_4_	α_5_	α_6_	α_6a_	α_6b_
1	1	0	0	0	1	0	0	0	0	1	0	0	1	0	0	0	0	1	0	0	0	1	1	0
2	1	0	0	0	1	0	0	0	0	1	0	0	1	0	0	0	0	1	0	0	1	0	0	0
3	1	0	0	0	1	0	0	0	0	1	0	0	1	0	0	0	1	0	0	0	1	0	0	0
4	0	1	0	0	0	1	1	0	1	0	0	0	1	0	0	0	1	0	0	0	1	0	0	0
5	0	0	1	0	1	0	0	0	1	0	0	0	0	1	0	1	0	0	1	0	1	0	0	0
6	0	1	0	0	1	0	0	0	0	0	1	0	1	0	0	0	0	0	1	0	1	0	0	0
7	0	0	1	0	1	0	0	0	0	0	1	0	1	0	0	0	1	1	0	0	0	1	0	1
8	0	0	1	0	1	0	0	0	0	0	0	1	1	0	0	0	0	1	0	0	0	1	0	1
9	1	0	0	0	1	0	0	0	1	0	0	0	0	1	1	0	0	0	1	0	1	0	0	0
10	0	0	0	1	1	0	0	0	0	1	0	0	1	0	0	0	0	0	0	1	1	0	0	0
11	0	0	1	0	1	0	0	0	1	0	0	0	1	0	0	0	0	0	0	1	1	0	0	0
12	0	0	0	1	1	0	0	0	0	0	0	1	1	0	0	0	0	0	0	1	1	0	0	0
13	0	0	0	1	1	0	0	0	0	0	1	0	1	0	0	0	0	0	1	1	1	0	0	0
14	0	1	0	0	0	1	0	1	0	0	0	1	1	0	0	0	0	1	0	0	1	0	0	0
15	0	0	0	1	0	1	0	1	0	0	1	0	0	1	1	0	0	0	0	1	1	0	0	0
16	0	1	0	0	1	0	0	0	0	0	0	1	1	0	0	0	1	0	0	0	1	0	0	0
Total	4	4	4	4	13	3	1	2	4	4	4	4	13	3	2	1	4	5	4	5	13	3	1	2

α_1_ = Retrieving information; α_2_ = Making inferences; α_3_ = Integration and summation; α_4_ = Reflective evaluation; α_5_ = Literary text; α_6_ = Practical text; α_6a_ = Expository text; α_6b_ = Discontinuous text.

### Measures

#### The Diagnostic Chinese Reading Comprehension Assessment (DCRCA)

DCRCA was developed as a multiple-choice, computer-based, online reading comprehension assessment to identify cognitive processes used during understanding literacy or practical short passages. The final DCRCA for grades 2 to 6 comprises 3 booklets, and each booklet contains 16 items. These items required students to answer multiple-choice questions on their comprehension of short passages. Students’ responses were scored dichotomously (0 = incorrect, 1 = correct) for each item. As already described, each item was intentionally constructed by experts to align with precisely one of the four processes of reading comprehension (α_1_–α_4_) and one text-related attribute (α_5_–α_6_). The total testing frequencies of the attributes were similar in the three final booklets, while the short passages in the three booklets were compiled with increased complexity. Cronbach’s α values for the assessment of the three booklets were 0.82, 0.71, and 0.64.

#### The Chinese Word Recognition Assessment

The Chinese word recognition assessment was adopted for validation purposes, and it was adapted from the Chinese character recognition task (Li et al., 2012) to measure students’ word recognition skills. Students listened to the sound of a word composed of a given Chinese character and then chose the correct character from three distracting character options. A total of 150 character items were collected based on Chinese language textbooks (Shu et al., 2003). The maximum score of this assessment was 150. The internal reliability of the assessment was 0.91.

### Sample

The study was conducted for a regional reading education project in Changchun City, China. The project aims to investigate the development of primary students’ reading ability, recommend books suitable for reading, and provide them with corresponding reading courses. A total of 21,466 grade 2 to grade 6 students from 20 primary schools completed the assessments in November 2020, accounting for 94.1% of the total sample. Students were aged from 7.3 to 13.2 years, and the proportion of male students was 52.4% in total.

### Procedure

Considering the large number of students participating in the DCRCA, the organization and implementation were completed by Chinese teachers and computer teachers of each class. Researchers trained all teachers and provided them with standardized assessment manuals. The assessments were administered collectively *via* an online web page, which presented one item at a time to students. The web page set all items as compulsory, so there was no missing value in the formal test as long as the student submitted successfully. Considering primary students’ computer proficiency, students only needed to click medium-size options with mice to answer all questions. Students took approximately 20 min to successively complete the test battery, including the Chinese Word Recognition Assessment and the DCRCA. All students received an assessment analysis report with a recommended reading list and learning suggestions 1 month after the testing.

### Analysis

Data were analyzed using R studio (R Core Team, 2021). As a correctly specified Q-matrix is considered a prerequisite of model-data fitness and low bias in diagnostic classifications (Rupp and Templin, 2008; Kunina-Habenicht et al., 2012), both theoretical and empirical procedures (de la Torre and Chiu, 2016) were applied iteratively to obtain the best attribute numbers and the best item-attribute relationships using the “GDINA” package, version 2.8.0 (Ma and de la Torre, 2020). The “CDM” package, version 7.5–15, was used for fitting CDMs (e.g., DINA, DINO, R-RUM, A-CDM, and G-DINA) based on the MMLE/EM algorithm (George et al., 2016; Robitzsch and George, 2019). The CDM package allows the estimation of rich sets of models, fit indices, and diagnostic validity with various emphases, which can help researchers find the most appropriate model. Two-parameter logistic item response theory (2PL-IRT) statistics were calculated using the ltm package (Rizopoulos, 2006).

## Results

### Q-Matrix Validation

Three types of Q-matrices were created for each booklet to evaluate the applicability of attributes. Q1 contained only the four commonly agreed-upon cognitive attributes (α_1_–α_4_), Q2 added two text-type attributes (α_5_ and α_6_) to Q1 with reference to the curriculum criterion, and Q3 added three text-type attributes (α_5_, α_6a_, and α_6b_) to Q1 with reference to PISA and PIRLS. These Q-matrices were compared based on the model-data fit of the G-DINA model and likelihood ratio test (see Table 3).

**Table 3:** Model-data fitting results for Q-matrix validation.

Booklet	Q-matrix	Npars	Relative fit			Absolute fit		
			-2LL	AIC	BIC	SRMSR	max χ^2^	*p* (max χ^2^)
KS1	Q1^a^	45	−38109.4	76308.7	76594.7	0.041	219.23	<0.001
	Q2^b^	90	−37547.7	75275.5	**75847.4**	0.016	11.30	0.09
	Q3^c^	97	−37540.7	75275.4	75891.8	0.015	9.32	0.27
	**Sug Q2** ^c^	94	**−37537.9**	**75263.8**	75861.2	0.015	7.45	0.76
KS2	Q1^a^	43	−86989.8	174065.6	174370.0	0.021	26.65	<0.001
	**Q2** ^**b**^	86	**−86569.9**	**173311.7**	**173920.5**	0.012	8.92	0.339
	Q3^b^	93	−86570.9	173327.8	173986.1	0.012	8.16	0.514
KS3	Q1^a^	47	−85552.4	171198.8	**171529.2**	0.015	44.05	<0.001
	**Q2** ^c^	94	**−85370.6**	**170929.2**	171590.0	0.011	5.84	1
	Q3^b^	101	−85371.6	170945.2	171655.3	0.010	6.00	1

Different letter superscripts in column 2 indicate significant model fit improvement between adjacent Q-matrices by likelihood ratio test within each booklet. The best relative fit results within each booklet are shown in bold.

The SRMSR values of all Q-matrices were acceptable (below the 0.05 rule of thumb suggested by Maydeu-Olivares, 2013), while none of Q1 could be accepted based on the max χ^2^. The -2LL and AIC values suggested a direction of improvement from Q1 to Q2, while the fit values of Q2 and Q3 were close in all booklets. Likelihood ratio tests were adopted between the adjacent Q-matrices within each booklet. We found that (1) all Q2 and Q3 values were significantly better than Q1 values (*p* < 0.001); (2) the -2LL and AIC differences between Q2 and Q3 were small and unstable, as *p* values fluctuated around significance boundaries for booklets KS1 to KS3 (*p* ≈ 0.006, 1.00 and 0.049 respectively); and (3) the BIC consistently favored Q2 over Q3, as it was more compact and efficient. In summary, the fit indices showed similarities across booklets, suggesting that the attribute structure was the same across key stages. Based on the above results, we chose Q2 as a basis to finalize the item-attribute relationship.

An empirical Q-matrix validation procedure was conducted on all Q2s to compare the proportion of variance accounted for (PVAF) by plausible q-vectors for a given item (de la Torre and Chiu, 2016). A given q-vector was deemed correct if it was the simplest vector with a PVAF above 0.95. The validation results suggested no modification for booklet KS2 or KS3 and generated suggested Q-vectors for items 6 and 15 in booklet KS1. This indicated a relatively high attribute-wise agreement between the provisional and data-driven Q-matrices across all booklets. After expert revisions and iterative modeling, researchers concluded that the suggested changes in the Q-matrix were consistent with what the item truly assessed. The likelihood ratio test suggested that the fit of finalized Q2 was significantly better than that of the initial Q2 and was slightly better than that of Q3 for booklet KS1. The final Q-matrices are given in Table 4.

**Table 4:** Final Q-Matrices.

Item	Booklet KS1						Booklet KS2						Booklet KS3					
	α_1_	α_2_	α_3_	α_4_	α_5_	α_6_	α_1_	α_2_	α_3_	α_4_	α_5_	α_6_	α_1_	α_2_	α_3_	α_4_	α_5_	α_6_
1	1	0	0	0	1	0	0	1	0	0	1	0	0	1	0	0	0	1
2	1	0	0	0	1	0	0	1	0	0	1	0	0	1	0	0	1	0
3	1	0	0	0	1	0	0	1	0	0	1	0	1	0	0	0	1	0
4	0	1	0	0	0	1	1	0	0	0	1	0	1	0	0	0	1	0
5	0	0	1	0	1	0	1	0	0	0	0	1	0	0	1	0	1	0
6	0	1	0	1*	1	0	0	0	1	0	1	0	0	0	1	0	1	0
7	0	0	1	0	1	0	0	0	1	0	1	0	1	1	0	0	0	1
8	0	0	1	0	1	0	0	0	0	1	1	0	0	1	0	0	0	1
9	1	0	0	0	1	0	1	0	0	0	0	1	0	0	1	0	1	0
10	0	0	0	1	1	0	0	1	0	0	1	0	0	0	0	1	1	0
11	0	0	1	0	1	0	1	0	0	0	1	0	0	0	0	1	1	0
12	0	0	0	1	1	0	0	0	0	1	1	0	0	0	0	1	1	0
13	0	0	0	1	1	0	0	0	1	0	1	0	0	0	1	1	1	0
14	0	1	0	0	0	1	0	0	0	1	1	0	0	1	0	0	1	0
15	1*	0	0	1	0	1	0	0	1	0	0	1	0	0	0	1	1	0
16	0	1	0	0	1	0	0	0	0	1	1	0	1	0	0	0	1	0
Total	5	4	4	5	13	3	4	4	4	4	13	3	4	5	4	5	13	3

α_1_ = Retrieving information; α_2_ = Making inferences; α_3_ = Integration and summation; α_4_ = Reflective evaluation; α_5_ = Literary text; α_6_ = Practical text. The Q-matrix modifications were denoted by *.

### Model Comparison

To select the optimal CDM for the whole assessment and to reveal the relationships among reading attributes, we compared five representative CDMs including DINA, DINO, R-RUM, A-CDM, and G-DINA models, for each booklet using the final Q-matrices. As Table 5 shows, the five CDMs performed stably across booklets. The AIC and -2LL values for the G-DINA models were the lowest in the three booklets, followed by the A-CDM and the R-RUM models, while the values of the more parsimonious DINO and DINA models were observably worse. The BIC favored A-CDM, G-DINA, and A-CDM in booklets KS1 to KS3. Likelihood ratio tests suggested that none of the other CDMs fit as good as the G-DINA model. For the absolute fit values, the SRMSR values of all CDMs were below 0.05. However, only the G-DINA had insignificant max χ^2^ values in all cases, indicating a good fit to the data, while the DINO and DINA models were stably rejected by the significance of max χ^2^ in all cases. It is evident that the G-DINA model fits the entire assessment data reasonably better than the more parsimonious reduced models.

**Table 5:** Model fit comparison of CDMs using the final Q-matrices.

Booklet	CDM	Npars	Relative fit			Absolute fit		
			-2LL	AIC	BIC	SRMSR	max χ^2^	*p* (max χ^2^)
KS1	DINA^a^	54	−38147.9	76403.8	76747.0	0.042	269.83	<0.001
	DINO^b^	54	−38101.0	76310.0	76653.2	0.040	225.18	<0.001
	RRUM^c^	72	−37600.7	75345.3	75802.9	0.019	11.56	0.08
	A-CDM^c^	72	−37592.3	75328.6	**75786.1**	0.017	10.51	0.14
	G-DINA^d^	94	**−37537.9**	**75263.8**	75861.2	0.015	7.45	0.76
KS2	DINA^a^	54	−86967.4	174042.8	174425.0	0.020	24.75	<0.001
	DINO^a^	54	−86992.3	174092.6	174474.8	0.021	24.27	<0.001
	RRUM^b^	70	−86653.9	173447.8	173943.3	0.013	13.97	0.02
	A-CDM^b^	70	−86675.2	173490.5	173986.0	0.018	39.49	<0.001
	G-DINA^c^	86	**−86569.9**	**173311.7**	**173920.5**	0.012	8.92	0.339
KS3	DINA^a^	54	−85647.9	171403.7	171783.4	0.017	21.80	<0.001
	DINO^b^	54	−85641.5	171391.0	171770.7	0.017	26.65	<0.001
	RRUM^c^	72	−85520.2	171184.3	171690.5	0.014	10.51	0.14
	A-CDM^d^	72	−85484.2	171112.3	**171618.5**	0.012	9.38	0.26
	G-DINA^e^	94	**−85424.1**	**171036.2**	171697.0	0.011	8.59	0.41

Different letter superscripts in column 2 indicate significant model-fit improvement among CDMs by likelihood ratio test within each booklet. The best relative fit results within each booklet are shown in bold.

### Reliabilities and Validity

Pattern accuracy (Pa) and pattern consistency (Pc) indices show the degree to which the examinees were accurately and consistently classified as masters and non-masters (Cui et al., 2012). Therefore, they were adopted as indicators of reliability in Table 6. The Pa values for each separate attribute were between 0.68 and 0.95, and the Pc values were between 0.63 and 0.92. Despite a lack of consensus on general guidelines for what constitutes a high or acceptable reliability (Templin and Bradshaw, 2013), these results indicated an above acceptable capacity of measuring students’ reading attributes.

**Table 6:** Mastery classification reliability.

Attributes	Booklet KS1		Booklet KS2		Booklet KS3	
	Pa	Pc	Pa	Pc	Pa	Pc
α_1_	0.86	0.81	0.93	0.90	0.71	0.63
α_2_	0.90	0.86	0.89	0.88	0.80	0.78
α_3_	0.90	0.86	0.93	0.90	0.70	0.63
α_4_	0.91	0.88	0.92	0.87	0.68	0.63
α_5_	0.95	0.92	0.83	0.74	0.86	0.82
α_6_	0.86	0.85	0.87	0.88	0.77	0.85

**Figure 2:**
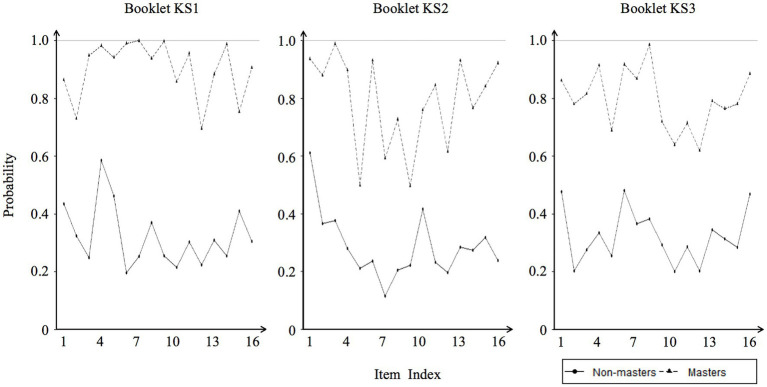
Item mastery plots.

Evidence of internal validity was provided using item mastery plots to quantify the various discriminatory and diagnostic capacities of test items (Roussos et al., 2007; von Davier and Lee, 2019). Figure 2 shows the item correct proportions for the masters versus the non-masters. The average item proportion correct difference was 0.53, and the differences in 41 out of the 48 items were greater than 0.40. This high value indicates a good fit between models and data, suggesting a strong diagnostic power of items and the DCRCA. In addition, this provided a valuable tool for finding poor items. For example, the differences of items 5 and 9 in booklet KS2 were smaller than 0.30. An in-depth examination suggested that these items were difficult; therefore, the item proportion correct for masters tended to be close to that for non-masters.

To further verify the external validity, the correlations between the scores on the DCRCA and the Chinese word recognition test were calculated. Word recognition scores were positively correlated with reading scores [KS1, *r* (4251) = 0.69, *p* < 0.001, KS2, *r* (8863) = 0.65, *p* < 0.001, KS3, *r* (8352) = 0.57, *p* < 0.001]. To summarize, the results suggested that the reliability and validity of the DCRCA were satisfactory.

### Skill Profiles

CDMs classify test-takers into latent classes, which represent skill mastery/non-mastery profiles for attributes specified in the Q-matrix. With the six-attribute Q-matrix structure, 64 theoretically existing latent classes (2^k^) were identified. For space considerations, only 15 skill profiles of the grade 2 students are presented in Table 7, as 49 classes showed lower posterior probabilities than 0.1%, suggesting that these skill classes may not be relevant to the data. Among the remaining 15 classes, the latent class [111111], mastery of all the subskills, had the highest posterior probability, followed by [000000], mastery of none of the subskills. CDM revealed that other dominant latent classes were [000011] and [111100], to which 27.15% of the test-takers belong. The profile [000011] might reflect children’s knowledge and experiences in reading specific text genres in the given items, while the profile [111100] might reflect children’s skills and experiences in answering specific reading tasks. This result supported the RAND report (RAND Reading Study Group, 2002) that mastery of the first four cognitive attributes and the last two text attributes may be relatively independent sources of variance in different reading comprehension scores.

**Table 7:** Latent classes and posterior probabilities.

#	Latent class	Posterior probability (%)	#	Latent class	Posterior probability (%)
1	111111	32.84	9	111000	0.56
2	000000	26.14	10	011111	0.52
3	000011	19.85	11	101100	0.45
4	111100	7.29	12	111010	0.40
5	100000	4.47	13	011011	0.26
6	010011	2.68	14	110000	0.21
7	110011	2.21	15	101000	0.16
8	111011	1.48		Total	99.53

## Discussion and Conclusion

This study developed and validated an instrument for diagnosing the strengths and weaknesses of Chinese reading comprehension ability at the primary level. Due to the criticism about a lack of true CDA research for educational purposes, the DCRCA was designed to meet the requirements of the Chinese curriculum criterion under the CDA framework proposed by Ravand and Baghaei (2020). Multiple steps were applied to maximize the diagnostic capacity and effectiveness of the DCRCA, including (1) gathering information about previous reading models and assessments; (2) specifying attribute lists based on the literature, student think-aloud protocols and expert review; (3) standardized test development and pilots; (4) empirical comparisons and refinements of Q-matrices and CDMs; and (5) reliability and validity analyses using the formal test data. The results indicate that the overall quality of the DCRCA is satisfactory and that the diagnostic classifications are reliable, accurate, and valid.

Following multiple procedures of attribute specification, model-data fit comparison, and empirical validation, the Q-matrix construction results yielded six final reading attributes, including four cognitive attributes that are consistent with cognitive processing and previous empirical studies of reading and two text-related attributes that were synthesized from large-scale assessment frameworks and the Chinese curricular criterion. Adding text-related attributes significantly improved the model-data fits of Q-matrices, implying that pragmatic or background knowledge of different text types might be vital in successful reading. The literacy text attribute is consistent with previous research, while the practical text attribute is a newly extracted attribute in CDM studies on reading. Our attempts to combine expository text with discontinuous text attributes may reveal their similarity in reading strategies and worth further investigation. The validation of text-related attributes also improved the application value and scope of the DCRCA because these attributes come from the experiences of educators and thus might be easier to recognize and train (Perfetti et al., 2005). Besides, the six-attribute structure has been scrutinized as a theoretical framework of reading comprehension for students at different developmental stages. This result provides evidence regarding the construct of primary-level Chinese reading and the DCRCA from theoretical and empirical perspectives.

The selection of the CDMs is critical in all CDA studies, as the optimal model not only caters to the diagnostic demands of the assessment but also reveals the interrelationships of attributes in the given domain. Five representative CDMs were compared, and the superiority of the G-DINA model was supported by all booklets and model-data fit. Therefore, it is safe to analyze the DCRCA with the saturated G-DINA model, which appeared to be flexible in accommodating various relationships among reading skills (Chen and Chen, 2016; Li et al., 2016; Ravand, 2016). The A-CDM model performed the closest level of fit indices to the G-DINA model. From a theoretical perspective, the A-CDM model could be a special case of the G-DINA model by only estimating the main effects of attributes, as the difference between the two models is that G-DINA allows additional estimation of interactions among latent skills (de la Torre, 2011). Therefore, given that the majority of the DCRCA items were designed to map one of the cognitive processes and one text type of reading, our findings support Stanovich’s (1980) interactive view of reading that holds both cognitive processes and text-related attributes to be crucial and interactive in successful execution of reading comprehension.

In addition, our results showed that the absolute fit indices preferred neither compensatory (A-CDM and DINO) nor non-compensatory (R-RUM and DINA) types of CDM, and max χ^2^ rejected all the reduced models in booklet KS2. Consequently, current results are not enough to assert that the relationship of reading attributes is either compensatory or non-compensatory. This is consistent with the findings of Jang (2009), Li et al. (2016), and Javidanmehr and Sarab (2019), who also voted for the co-existence of compensatory and non-compensatory relationships among the latent reading subcomponents.

The present study examined the diagnostic reliability and validity of the DCRCA. Reliability evidence is generally considered essential support for interpreting test results. The pattern accuracy and consistency index (Cui et al., 2012) suggested that the DCRCA reliably measures multiple reading attributes. Validity analyses are rarely conducted, with less than 22% of studies providing such information according to the literature review (Sessoms and Henson, 2018). Therefore, construct, internal, and external validities are provided for the Q-matrix and the DCRCA. The Q-matrix validation results suggest that the provisional Q-matrices have an approximately 95% attribute-wise agreement rate across booklets, which provides strong evidence for the construct validity of Q-matrix constructions (Deonovic et al., 2019). The internal validity evidence showed that the average proportion correct differences for each item were sufficiently large for most of the test items, indicating that these items have satisfying diagnostic capacity to differentiate masters from non-masters of reading. The mean score differences of only 4% of the items were less than 0.3, much lower than the proportion of 23% in retrofitted studies (Jang, 2009). This might be because retrofitting studies had to include many items that were weakly associated with targeted attributes. The possible presence of nondiagnostic items could lead to critical issues in the validity of measures of skill competencies, and thus, the test inferences might be limited.

The present study contributes to instructional practices at the elementary school level, as the assessment can provide reliable, valid and useful diagnostic information. This is the first empirical study that attempts to provide evidence in construct invariance of diagnosing Chinese reading attributes at different primary grades. As reading assessment can function as formative assessment, such diagnostic feedback could be further utilized by teachers and educators for monitoring learning progressions, providing remedial instructions for reading courses and programs. However, some limitations are also worth enumerating. First, the present research did not examine how diagnostic feedback is perceived and utilized by students and teachers in a classroom setting. More studies are needed to reveal the influences of CDA applications. Second, as the DCRCA was not equated vertically, the attribute mastery states can be compared only within each key stage. Future studies are needed to apply appropriate longitudinal CDMs (Zhan et al., 2019) or vertical equating methods (von Davier et al., 2008) for CDA to investigate the developmental course of students’ reading attributes. Third, the present study did not include a sufficient number of items to assess attributes a_6a_ and a_6b_. The Q-matrices may not be exhaustive to capture all reading comprehension and likely lead to limitations of the present study. Therefore, caution should be taken in interpreting our final results, and explorations of a more balanced Q-matrix construction are needed in the future. Last, although the results related to model fit and item parameters were fairly acceptable, future research should seek to improve the psychometric properties to make the report inferences more reliable. Therefore, the study was only a start. A deeper understanding of CDM application may be deduced by interpreting the dominant skill classes as learning states and the combination of skill classes as learning paths and learning progressions (Wu et al., 2020). Future studies are needed to help instructors design suitable learning plans with fine-grained diagnostic reports of students. In addition, more well-designed items can be generated and scaled as formative and summative assessments to satisfy expectations from the curriculum criterion. With the help of the DCRCA, teachers could design their own classroom reading materials and assessments as learning objectives that they wish students to attain.

## Data Availability Statement

The raw data supporting the conclusions of this article will be made available by the authors, without undue reservation.

## Ethics Statement

The studies involving human participants were reviewed and approved by Ethics and Human Safety Committee, Faculty of Psychology, Beijing Normal University. Written informed consent to participate in this study was provided by the participants’ legal guardian/next of kin.

## Author Contributions

YL and JL conceived the study. YL and MZ organized the pilots and analyzed the original data. MZ developed the test items and conducted the think-aloud protocols. YL collected the formal test, analyzed the data, and wrote the manuscript. JL provided technical advices. All authors contributed to the article and approved the submitted version.

## Funding

This work was supported by the National Natural Science Foundation of China (31861143039) and the National Key R&D Program of China (2019YFA0709503).

## Conflict of Interest

The authors declare that the research was conducted in the absence of any commercial or financial relationships that could be construed as a potential conflict of interest.

## Publisher’s Note

All claims expressed in this article are solely those of the authors and do not necessarily represent those of their affiliated organizations, or those of the publisher, the editors and the reviewers. Any product that may be evaluated in this article, or claim that may be made by its manufacturer, is not guaranteed or endorsed by the publisher.

## Supplementary Material

The Supplementary Material for this article can be found online at: https://www.frontiersin.org/articles/10.3389/fpsyg.2021.786612/full#supplementary-material
