# Supplementary material for: Validating a Reading Assessment Within the Cognitive Diagnostic Assessment Framework: Q-Matrix Construction and Model Comparisons for Different Primary Grades
Source: Front Psychol. 2021 Dec 16;12:786612. doi: 10.3389/fpsyg.2021.786612 (PMC8716379; doi:10.3389/fpsyg.2021.786612)
Supplement: Supplementary file 1 [file Table_1.DOCX]

**Supplementary Table 1. Cognitive attributes of reading comprehension.**

| **Test** | **Author** | **Age** | **Language Background** | **Attribute Number** | **Common Attributes** | | | | | | **Specific Attributes** |
| --- | --- | --- | --- | --- | --- | --- | --- | --- | --- | --- | --- |
| PIRLS | (Yun, 2017) | Grade 4 | Native | 6 |  | Retrieval | Inference | Integration | Evaluation |  | Constructed response; Expository text |
| PIRLS | (George & Robitzsch, 2021; Toprak-Yildiz, 2021) | Grade 4 | Native | 4 |  | Focus on & retrieve explicitly stated information | Make straightforward  inferences | Interpret &  integrate  ideas and  information | Evaluate &  critique  content  and textual  elements |  |  |
| Reading Achievement Measure | (Jang et al., 2015) | Grade 5–6 | Diverse backgrounds | 6 | Processing vocabulary knowledge | Textually explicit comprehension | Textually implicit comprehension; Inferencing | Summarizing main ideas in text |  | Processing grammatical knowledge |  |
| Reading Comprehension Assessment of Modern Chinese Prose | (Xie, 2014) | Grade 7-9 | Native Chinese | 7 | Word decoding | Information extraction | Information deduce | Content analysis; Content generalization | Text evaluation |  | Formal schema |
| PISA | (Chen & de la Torre, 2014) | 15 y | Native | 5 |  | Locating information | Developing a logical interpretation | Forming a broad general understanding | Evaluating the quality or appropriateness of a text |  | Evaluating a number-rich text with number sense |
| PISA | (Chen & Chen, 2016) | 15 y | Native | 7 | Interpreting conceptual meanings | Identifying explicit information | Making inferences | Generalizing main ideas | Evaluating and commenting |  | Understanding charts and graphs; Expressing in written forms |
| the CDSLRCT | (Toprak & Cakir, 2021) | 18–23 y | EFL | 5 |  | Understanding  explicit information | Inferencing | Using discourse knowledge | Recognizing mood, purpose, attitude, and tone of the author | Using syntax knowledge |  |
| the INUEE | (Ravand, 2016; Ravand & Robitzsch, 2018) | 22–25 y | EFL | 5 | Vocabulary | Detail | Inference | Main idea |  | Syntax |  |
| MELAB | (Sawaki, Kim & Gentile, 2010; Li, 2011; Javidanmehr & Sarab, 2019) | Adults | Diverse backgrounds | 5 | Vocabulary | Extracting explicit information | Making inferences | Connecting and synthesizing |  | Syntax |  |
| MELAB | (Li & Suen., 2013) | Adults | EFL | 4 | Vocabulary | Extracting explicit information |  | Connecting and synthesizing |  | Syntax |  |
| MELAB | (Li et al., 2016) | Adults | Diverse backgrounds | 4 | Vocabulary | Extracting  explicit information | Understanding  implicit information |  |  | Syntax |  |

**Note.** PIRLS: the Progress in International Reading Literacy Study; PISA: the Programme for International Student Assessment; TOEFL: the Test of English as a Foreign Language; the CDSLRCT: the Cognitive Diagnostic Second Language Reading Comprehension Test; MELAB: the Michigan English Language Assessment Battery; the INUEE: the Iranian National University Entrance Examination.

**Supplementary Table 2. The characteristics of the short texts in each booklet.**

| Booklet | Text Difficulty | Text length | Tokens | Lexical Difficulty | Function Word Ratio |
| --- | --- | --- | --- | --- | --- |
| KS1 | 3.38 | 150.60 | 81.20 | 2.60 | 25.0% |
| KS2 | 3.69 | 156.87 | 86.53 | 3.08 | 22.4% |
| KS3 | 4.40 | 278.57 | 131.93 | 3.03 | 25.7% |
| Average | 3.81 | 193.45 | 99.16 | 2.90 | 24.4% |

Note. The text characteristics were calculated with the Chinese readability formula, which was developed to link the average reading ability of primary students and the text difficulty levels of recommended reading materials for independent reading (Liu, Li, Wang, Gan & Li, 2021). The text difficulty value ranges from 1 to 12, indicating the semester level of the recommended books or textbook materials that most students of that semester can read fluently. The text difficulty value = -2.44 + 0.01 × tokens +1.03 × lexical difficulty + 9.32 × function word ratio. Tokens represent the type of Chinese characters in each text. Lexical difficulty reflects the average occurring levels of the non-repeated Chinese words in each text. In Chinese language, function words express the grammatical relationships of texts and have little semantic content of its own. The function word ratio is considered a proxy for sentence complexity, which is represented by the sum percentage of prepositions, conjunctions, modal particles, auxiliary words, adverbs and locatives in each text.
